# Dataset demonstrating effects of momentum transfer on sizing of current collector for lithium-ion batteries during laser cutting

**DOI:** 10.1016/j.dib.2017.12.021

**Published:** 2017-12-19

**Authors:** Dongkyoung Lee, Jyotirmoy Mazumder

**Affiliations:** a Department of Mechanical and Automotive Engineering, Kongju National University, Cheonan 31080, Republic of Korea; b Center for Lasers and Plasmas for Advanced Manufacturing(CLPAM), Department of Mechanical Engineering, University of Michigan, Ann Arbor, MI 48109, USA; c Department of Material Science and Engineering, University of Michigan, Ann Arbor, MI 48109, United States

## Abstract

Material properties of copper and aluminum required for the numerical simulation are presented. Electrodes used for the (paper) are depicted. This study describes the procedures of how penetration depth, width, and absorptivity are obtained from the simulation. In addition, a file format extracted from the simulation to visualize 3D distribution of temperature, velocity, and melt pool geometry is presented.

**Specifications Table**

**Table:** 

Subject area	*Mechanical engineering, Manufacturing engineering, Applied physics, Computational Analysis*
More specific subject area	*Laser cutting, lithium-in battery manufacturing engineering*
Type of data	*Table, graph and figure*
How data was acquired	*Material properties are obtained from ref []. Raw data of numerical simulation are obtained by Fortran90. The raw data are filtered and analyzed by MATLAB. Filtered data are plotted with Tecplot*
Data format	*Raw, filtered, and analyzed*
Data source location	*Cheonan, South Korea*
Data accessibility	*Dataset is within this article*
Related research article	*Dongkyoung Lee, Jyotirmoy Mazumder, Effects of momentum transfer on sizing of current collector for lithium-ion batteries during laser cutting*[1]

**Value of the data**

• The summary of material properties can be easily accessed from the various applications since copper and aluminum are popular materials.
• Researchers could be referred to this dataset to design, compare, analyze, and validate another theoretical model of laser cutting on current collectors.
• Analyzing these data, one can compare and ensure the validity of experimental approaches and results.
• The values of the performance parameters can be used to compare the simulation result of laser cutting of current collector for lithium-ion batteries

## 1 Data

The material properties for current collector materials such as copper and aluminum used for the mathematical model have been presented in Table 1 and Table 2 respectively. All of these material properties are extracted from the published literatures [2], [3], [4], [5], [6], [7].

**Table 1:** Material properties of copper.

Property	Value
Melting temperature	1357.77(K)
Normal boiling temperature	2835.15(K)
Critical point temperature	8280(K)
Liquid density	7920(kg m^-3^)
Solid density	8960(kg m^-3^)
Kinematic viscosity	3.50E-07(m^2^ s^−^^1^) [2]
Surface tension	1.257-0.0002*(T-1356) (N m^−^^1^) [3]
Latent heat of vaporization	5.23E+06(J kg^−^^1^)
Latent heat of fusion	2.05E+05(J kg^−^^1^)
Solid thermal conductivity	317(W m^−1^ K^−^^1^) [4]
Liquid thermal conductivity	157(W m^−1^ K^−1^) [4]
Liquid constant-pressure specific heat	571.6218(J kg^−1^ K^−^^1^)
Solid constant-pressure specific heat	385(J kg^−^^1^ K^−^^1^) [5]
Liquid thermal diffusivity	3.62E-05(m^2^ s^−^^1^)
Solid thermal diffusivity	7.63E-05(m^2^ s^−^^1^)
Laser absorptivity for flat surface	0.05

**Table 2:** Material properties of aluminum.

Property	Value
Melting temperature	933.47(K)
Normal boiling temperature	2792(K)
Critical point temperature	7963(K)
Liquid density	2333(kg m^−3^)
Solid density	2700(kg m^−3^)
Kinematic viscosity	4.43635E-07(m^2^ s^−1^) [6]
Surface tension	0.860-0.000115*(T-933.47) (N m^−1^) [7]
Latent heat of vaporization	1.09E+07(J kg^−1^)
Latent heat of fusion	3.97E+05(J kg^−1^)
Solid thermal conductivity	237(W m^−^^1^ K^−1^)
Liquid thermal conductivity	93.752(W m^−1^ K^−1^)
Liquid constant-pressure specific heat	1255.2(J kg^−^^1^ K^−1^)
Solid constant-pressure specific heat	896.9607116(J kg^−1^ K^−^^1^)
Liquid thermal diffusivity	3.20E-05(m^2^ s^−1^)
Solid thermal diffusivity	9.79E-05(m^2^ s^−1^)
Laser absorptivity for flat surface	0.07

**Fig. 1:**
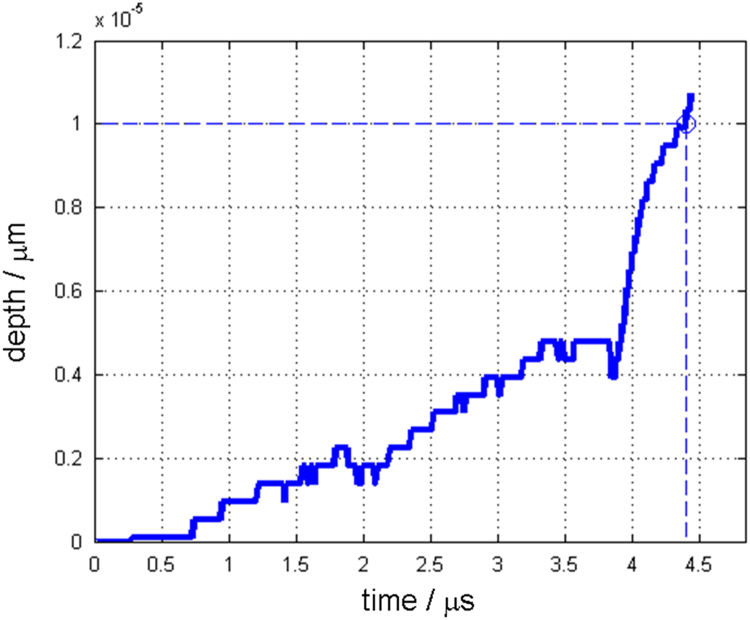
Penetration depth of copper with the laser power of 250 W and scanning speed of 3000 mm s^−1^.

**Fig. 2:**
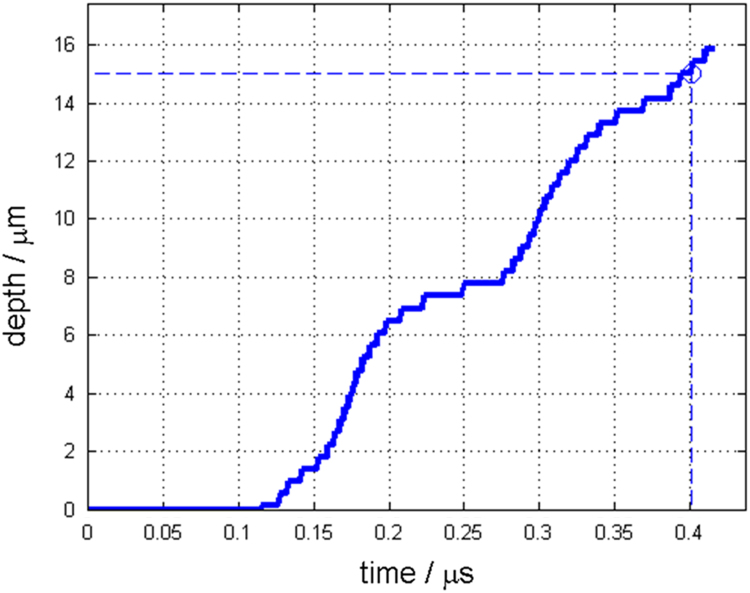
Penetration depth of aluminum with the laser power of 150 W and scanning speed of 3000 mm s^−1^.

**Fig. 3:**
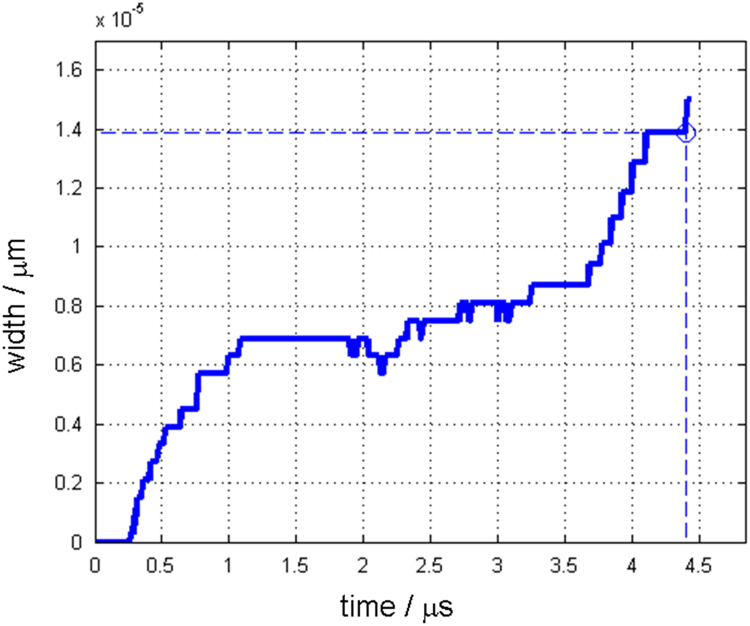
Kerf width of copper with the laser power of 250 W and scanning speed of 3000 mm s^−1^.

**Fig. 4:**
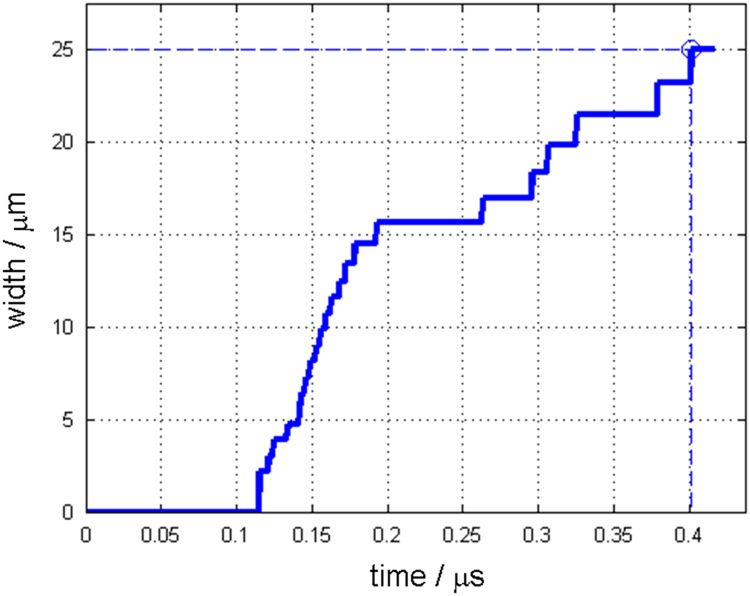
Ker width of aluminum with the laser power of 150 W and scanning speed of 3000 mm s^−^^1^.

**Fig. 5:**
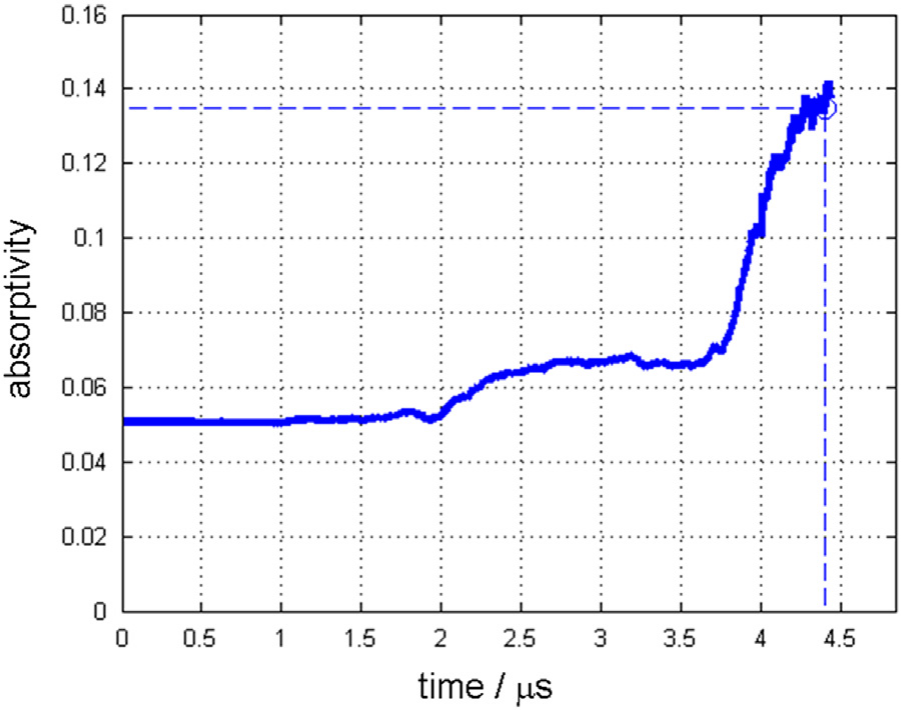
Absorptivity of copper with the laser power of 250 W and scanning speed of 3000 mm s^−1^.

**Fig. 6:**
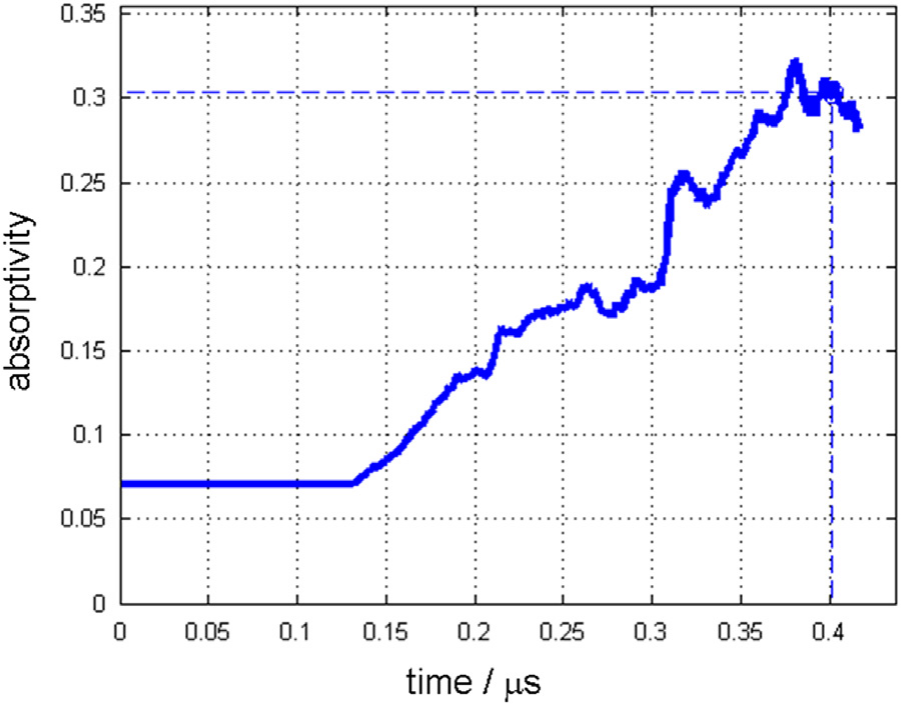
Absorptivity of aluminum with the laser power of 150 W and scanning speed of 3000 mm s^−1^.

Along with this dataset, the simulation parameters are tabulated in Table 3. Fig. 1 and Fig. 2 show depth changes during laser cutting of copper and aluminum depending on elapsed time, respectively. Depth values are measured from the material surface (*Z*=0) to the tip of penetration hole, which is the minimum Z value of the liquid/vapor interface coordinate(*Z*=min($\phi_{L/V}$)) [1]. Fig. 3 and Fig. 4 show kerf width changes during the laser cutting of copper and aluminum depending on elapsed time, respectively. Kerf width values are measured from the center of the laser beam to the maximum width of deep penetration in Y axis, which is the maximum Y value of the liquid/vapor interface coordinate (*Y*=max($\phi_{L/V}$)) [1]. Since, the proposed mathematical model [1] uses a symmetric coordinate, the attained kerf width values are doubled to fully represent the whole kerf width. Fig. 5 and Fig. 6 show absorptivity changes during laser cutting of copper and aluminum depending on elapsed time, respectively. Absorptivity is obtained as a ratio of an absorbed laser energy, after considering multiple reflections, to an irradiated laser energy.

**Table 3:** Simulation parameters.

Category	Value
Laser irradiation mode	Continuous
Laser beam wavelength	1070 (μm)
Laser beam diameter	11 (um)
Laser beam distribution	Gaussian
Mesh type	Non-uniform
Numerical domain	75 μm × 30 μm × 90 μm
Thickness of copper	10 (μm)
Thickness of aluminum	15 (μm)
Laser power of copper	250 (W)
Laser power of aluminum	150 (W)
Laser scanning speed	3 (m s^−^^1^)
Discretization of governing equations	Implicit finite difference method
Discretization of level set method	2nd order space convex scheme
Matrix solver	Conjugated Gradient Stabilized method
Coupled pressure-velocity solver	Semi-Implicit Method for Pressure-Linked Equation-Consistent

**Fig. 7:**
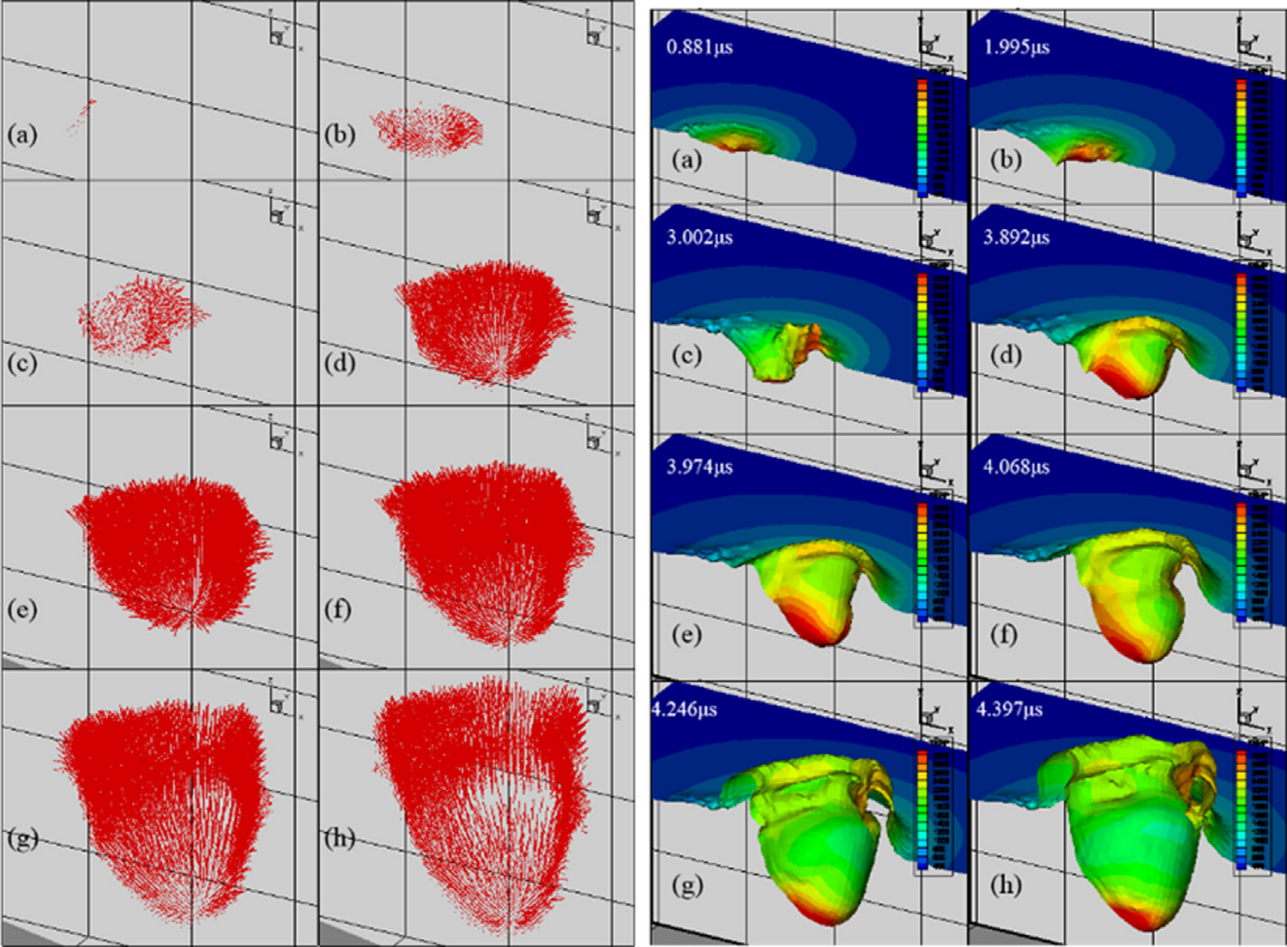
Melt pool flow (left) and Temperature distribution (right) of copper.

**Fig. 8:**
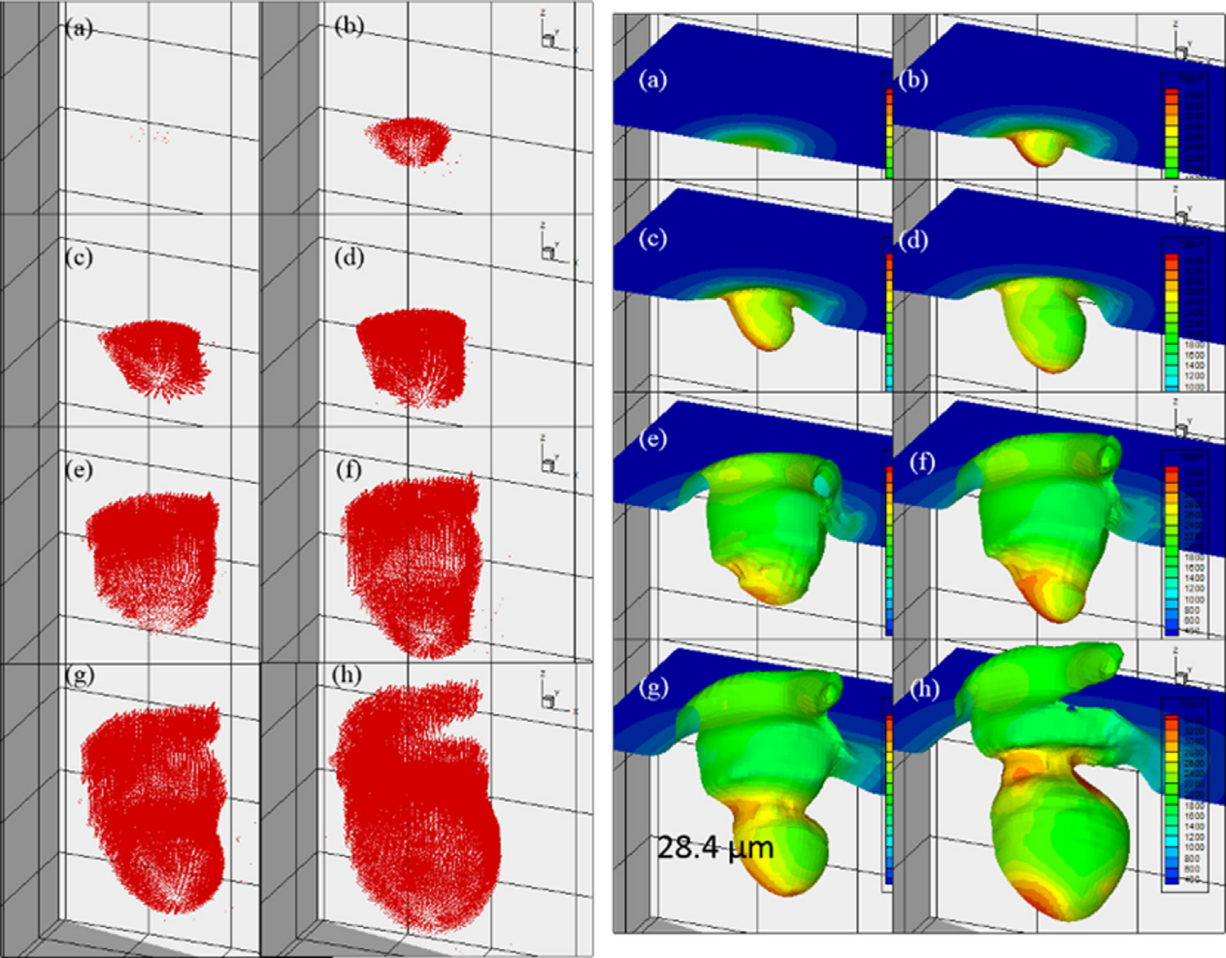
Melt pool flow (left) and Temperature distribution (right) of aluminum.

Fig. 7 and Fig. 8 show melt pool flow and temperature and distribution of copper and aluminum, respectively. From the dataset, the temperature and velocity values in an evaporated region are set to invisible only for a visualization purpose. The dataset is extracted from the simulation at each time. Carefully chosen dataset at specific time are plotted in 3D view. An appropriate angle to fully visualize characteristics of the temperature and melt pool flow is carefully chosen.

## 2 Experimental design, materials and methods

### 2.1 Simulation design

Since this paper includes no experiments, the simulation design is presented. To demonstrate physical phenomena with full penetration, simulation design is referred to the [8]. Among available laser parameters, the laser power of 250 W and laser speed of 3000 mm/s are chosen for copper as well as the laser power of 150 W and laser speed of 3000 mm/s are chosen for aluminum.

### 2.2 Materials

**Fig. 9:**
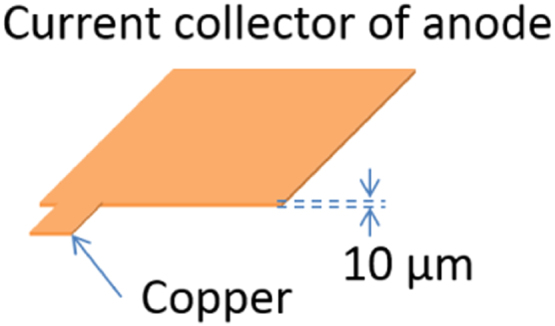
Material used for current collector of anode: copper.

**Fig. 10:**
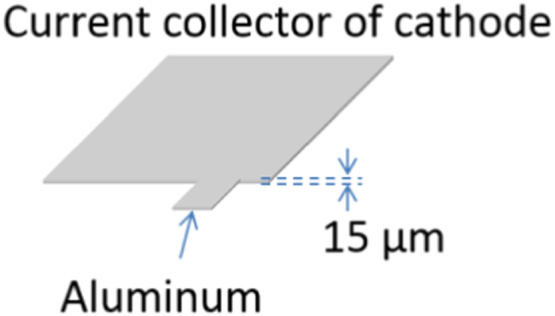
Material used for current collector of cathode: aluminum.

Materials used for current collectors of anode and cathode are shown in Fig. 9 and Fig. 10, respectively. The thickness of copper and aluminum are 10 μm and 15 μm, respectively. To the sake of simplicity, the thickness of commercially available copper and aluminum foils is used.

### 2.3 Methods

**Fig. 11:**
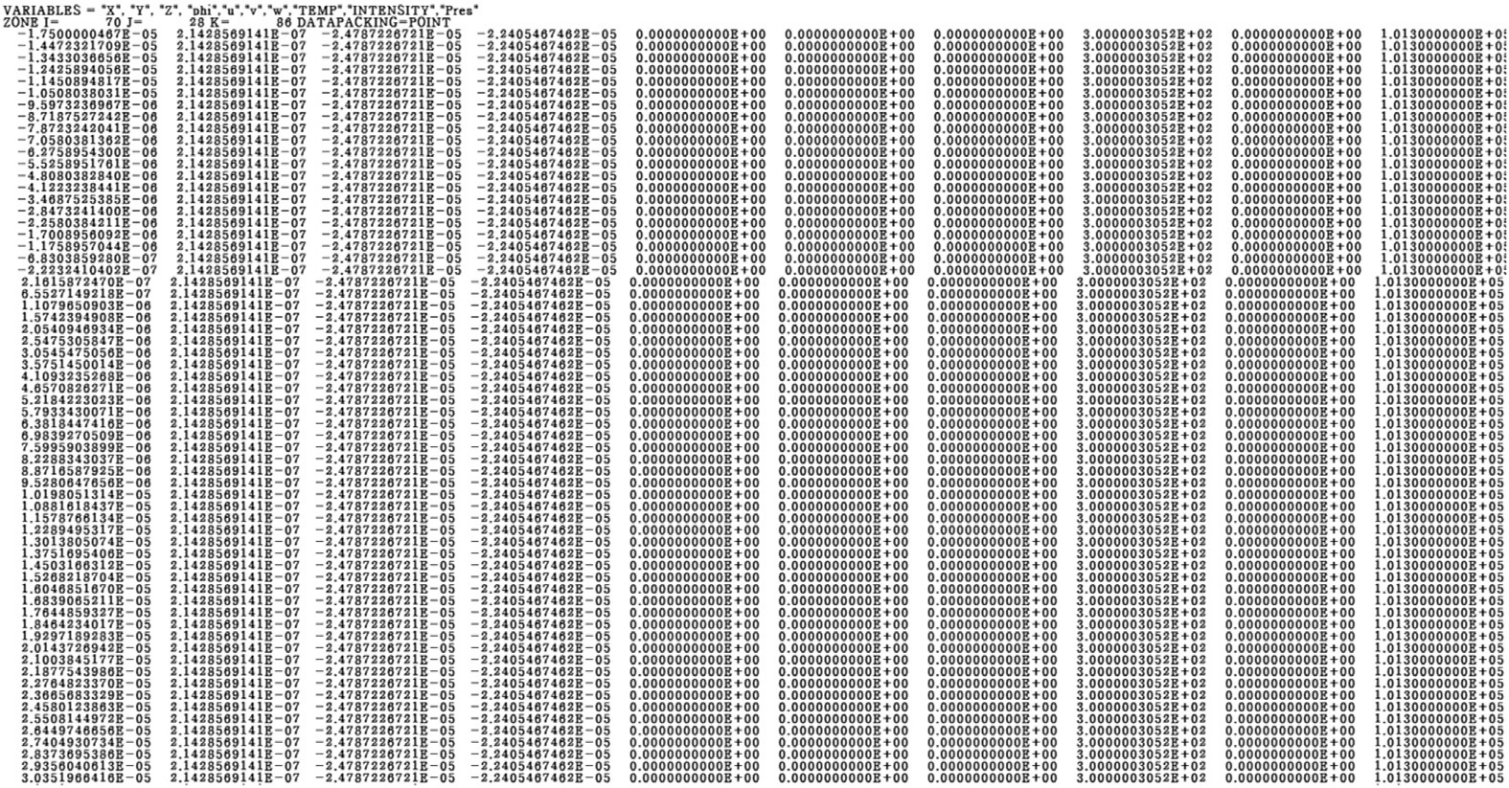
Tecplot format captured.

Dataset of penetration hole depth, width, and absorptivity are saved from the simulation for each time step. After the dataset is obtained, graph plotted by MATLAB code. To make a 3D plot for each time, velocity, temperature, and level set data are save in the form of Tecplot format. The Tecplot format captured is shown in Fig. 11.
