# Supplementary material for: Dataset demonstrating effects of momentum transfer on sizing of current collector for lithium-ion batteries during laser cutting
Source: Data Brief. 2017 Dec 19;17:6–14. doi: 10.1016/j.dib.2017.12.021 (PMC5767567; doi:10.1016/j.dib.2017.12.021)
Supplement: Supplementary file 1 — Supplementary material [file mmc1.docx]

**Title:** **Dataset demonstrating effects of momentum transfer on sizing of current collector for lithium-ion batteries during laser cutting**

The authors whose names are listed immediately below certify that they have NO affiliations with or involvement in any organization or entity with any financial interest (such as honoraria; educational grants; participation in speakers’ bureaus; membership, employment, consultancies, stock ownership, or other equity interest; and expert testimony or patent-licensing arrangements), or non-financial interest (such as personal or professional relationships, affiliations, knowledge or beliefs) in the subject matter or materials discussed in this manuscript.

**Corresponding Author names:**

**Dongkyoung Lee**
